# Rates and associations of relapse over 5 years of 2649 people with bipolar disorder: a retrospective UK cohort study

**DOI:** 10.1186/s40345-023-00302-x

**Published:** 2023-06-30

**Authors:** Danielle Hett, Isabel Morales-Muñoz, Buse Beril Durdurak, Max Carlish, Steven Marwaha

**Affiliations:** 1 Institute for Mental Health, University of Birmingham, Edgbaston, Birmingham, B15 2TT UK; 2 The Barberry National Centre for Mental Health, Birmingham and Solihull Mental Health Trust, Birmingham, B15 2SJ UK

**Keywords:** Relapse, Bipolar disorder, Naturalistic, Epidemiology

## Abstract

**Background:**

Evidence regarding the rate of relapse in people with bipolar disorder (BD), particularly from the UK, is lacking. This study aimed to evaluate the rate and associations of clinician-defined relapse over 5 years in a large sample of BD patients receiving routine care from a UK mental health service.

**Method:**

We utilised de-identified electronic health records to sample people with BD at baseline. Relapse was defined as either hospitalisation, or a referral to acute mental health crisis services, between June 2014 and June 2019. We calculated the 5-year rate of relapse and examined the sociodemographic and clinical factors that were independently associated with relapse status and the number of relapses, over the 5-year period.

**Results:**

Of 2649 patients diagnosed with BD and receiving care from secondary mental health services, 25.5% (n = 676) experienced at least one relapse over 5 years. Of the 676 people who relapsed, 60.9% experienced one relapse, with the remainder experiencing multiple relapses. 7.2% of the baseline sample had died during the 5-year follow-up. Significant factors associated with experiencing any relapse, after adjustment for relevant covariates, were history of self-harm/suicidality (OR 2.17, *CI* 1.15–4.10, *p* = 0.02), comorbidity (OR 2.59, *CI* 1.35–4.97, *p* = 0.004) and psychotic symptoms (OR 3.66, *CI*  1.89–7.08, *p* < 0.001). Factors associated with the number of relapses over 5 years, after adjustment for covariates, were self-harm/suicidality (*β* = 0.69, *CI* 0.21–1.17, *p* = 0.005), history of trauma (*β* = 0.51, *CI* = 0.07–0.95, *p* = 0.03), psychotic symptoms (*β* = 1.05, *CI* 0.55–1.56, *p* < 0.001), comorbidity (*β* = 0.52, *CI* 0.07–1.03, *p* = 0.047) and ethnicity (*β* = − 0.44, *CI* − 0.87 to − 0.003, *p* = 0.048).

**Conclusions:**

Around 1 in 4 people with BD in a large sample of people with BD receiving secondary mental health services in the UK relapsed over a 5-year period. Interventions targeting the impacts of trauma, suicidality, presence of psychotic symptoms and comorbidity could help to prevent relapse in people with BD and should be considered in relapse prevention plans.

**Supplementary Information:**

The online version contains supplementary material available at 10.1186/s40345-023-00302-x.

## Introduction

Bipolar disorder (BD) is often a debilitating mental illness, affecting approximately 1–2% of the population and is associated with poor quality of life, cognitive impairment, an increased risk of completed suicide and substantial financial costs to society (Vieta et al. 2018). Critical to this suffering and financial cost is the rate of relapse among this group. BD is marked by frequent mood episodes/relapses (i.e. manic, depressive and mixed), with each of these relapses having the potential to take years from—or even to destroy—a life. Understanding relapse over time to inform better shared decision-making for clinicians and patients is likely to be of enormous benefit for both, and for mental health services in general. Identifying factors associated with relapse also enables the identification of modifiable risk factors which can then form new targets for intervention and prevention efforts in BD. Further, patients deserve this information to make decisions about their own lives and care.

### Estimating BD relapse: intervention vs naturalistic studies

Estimated relapse rates within BD are largely based upon data from randomised controlled trials (RCTs), and although these studies are clinically important, they are limited by the selection bias commonly present in the patient recruitment of RCTs (e.g. excluding patients with more complex BD presentations such as comorbid diagnoses), and thus limit the generalisability of results to a wider BD population who typically have more complex BD presentations than those recruited in RCT’s. RCTs are also constrained by their duration; as most trials are conducted over a few weeks/months at most and research clearly demonstrates that BD is a lifelong journey (Vieta et al. 2018). Naturalistic studies offer approaches to addressing the biases associated with RCT’s, as they have strong ecological validity and may offer a more accurate representation of the longer-term outcome of those with BD by utilising ‘real-world data’. For instance, early naturalistic data shows the BD relapse rate to be as high as 67.5% during a 5-year period (n = 117) (Coryell et al. 1989). Further, one review paper calculated pooled relapse rates by RCT studies (N = 15) and naturalistic studies (n = 10), reporting relapse rates of 21.9% and 26.3% per year, respectively (Vázquez et al. 2015).

The EMBLEM Study (European Mania in Bipolar Longitudinal Evaluation of Medication) (Hong et al. 2010) appears to be the largest (N = 1379) naturalistic study examining relapse. This 2-year prospective study assessed relapse rates and the outcome of pharmacological treatment of manic and mixed BD episodes in BD from 14 European countries, including the UK (UK sample size not reported in the paper). They reported a relapse rate of 54.3% over 2 years, with relapse defined as at least a one-point increase in the Clinical Global Impression Bipolar Disorder scale score, inpatient admission or relapse determined after psychiatric assessment. Similar relapse rates (48.5%) were observed by Perlis et al.’s (2006) prospective study of patients (N = 858) enrolled in the US multicentre outpatient study; Systematic Treatment Enhancement Program for Bipolar Disorder (STEP-BD) (Perlis et al. 2006; Sachs et al. 2003). More recently, a 6-year naturalistic study based in Taiwan (N = 165) found that 77.6% of patients were readmitted to hospital by 6 years (Li et al. 2018).

Importantly however, these studies do not offer any insight into the rates of relapse within the UK, where unlike other countries, there is a well-developed model of community healthcare services free at the point of use. O’Hagan et al. (2017) appears to be the only UK-based naturalistic study to examine rates of BD relapse. Authors investigated a 1-year follow-up of patients (N = 519) discharged from a single inpatient mental health unit following BD treatment. The relapse rate was 32.2%, defined as a readmission to hospital within 1-year of discharge. In sum, the few studies to date which estimate the rate of BD relapse all are either based outside of the UK, are from RCTs or have small samples. Given the limits of the extant data, we aimed to investigate relapse over the medium term in people with BD and expand on previous work by examining 5-year relapse outcomes in BD patients receiving routine care from a large, diverse, UK-based mental healthcare Trust. Additionally, it is important to identify potentially modifiable risk factors associated with relapse status among BD populations. To date, the literature has identified several factors associated with BD relapse status, including age of BD onset (Gignac et al. 2015), gender (Degenhardt et al. 2012; Tundo et al. 2018), number of previous mood episodes (Tundo et al. 2018), history of psychotic symptoms (Carlson et al. 2012), comorbidity of mental health conditions (Perlis et al. 2006; Yen et al. 2016), rapid cycling (Degenhardt et al. 2012), greater depressive symptoms at baseline (Degenhardt et al. 2012), smoking (O’Hagan et al. 2017) and psychosocial stress (Dios et al. 2012).

## Study aims

1. To calculate the 5-year rate of relapse in patients with bipolar disorder receiving care from a UK secondary mental healthcare service.
2. To examine the socio-demographic and clinical factors associated with relapse status over 5 years.
3. To examine the associations of multiple relapses over 5 years.

## Method

### Study setting and data source

Birmingham and Solihull Mental Health NHS Foundation Trust (BSMHFT) serves a population of 1.3 million people, and currently offers mental health care to over 370,000 individuals. Electronic health records (EHRs) are routinely used throughout the Trust to document patient information and progress throughout the care system. The Clinical Record Interactive Search (CRIS) system is a research database that contains de-identified data from NHS Trusts’ EHRs.

We used the CRIS system to extract de-identified patient data from within BSMHFT. This database has approval for secondary analysis in line with BSMHFT’s research policies, and thus, according to the Trust’s policy, the current project did not require NHS Research Ethics approval. However, the study was fully reviewed and approved by the BSMHFT Research and Innovation Department prior to accessing the data. The available CRIS data used in the current analysis covered patients details and service use from June 2014 to June 2019. To identify the occurrence of relapse and calculate relapse rates over the 5-year period, we extracted clinical data via two sources: (1) structured data fields (e.g. demographic, diagnostic and referral data); and (2) free-text documents (e.g. clinical progress notes) (see *Measures* section below).

### Study cohort

The inclusion criteria for the study cohort were: (1) diagnosed with a bipolar affective disorder (according to the International Classification of Diseases version 10 [ICD-10] diagnostic codes F30 and F31 by June 2014; (2) aged at least 18 years old by June 2014; and.

(3) accessing secondary mental health services in the Trust at some point between June 2014 and June 2019. Please see Table 1 for a breakdown of the diagnostic categories of the full sample. As outlined in Table 1, one quarter (i.e. 26%) were coded as in remission and the rest were coded at study entry with a variety of other ICD-10 bipolar disorder diagnoses. However, we do not know whether these were current episodes due to the quality of the data.

**Table 1:** Diagnostic categories of the full cohort at entry (N = 2, 649)

ICD-10 diagnostic code at BD diagnosis date (i.e. earliest recorded date within data)	Diagnosis code description at BD diagnosis date (earliest recorded within data)	n (%) of full sample
F300	Hypomania	63 (2.38%)
F301	Mania without psychotic symptoms	16 (0.60%)
F302	Mania with psychotic symptoms	71 (2.68%)
F308	Other manic episodes	2 (0.08%)
F309	Manic episode, unspecified	11 (0.42%)
F310	Bipolar affective disorder current episode hypomanic	392 (14.80%)
F311	Bipolar affective disorder current episode manic, without psychotic symptoms	93 (3.51%)
F312	Bipolar affective disorder current episode manic, with psychotic symptoms	152 (5.74%)
F313	Bipolar affective disorder, current episode mild or moderate depression	517 (19.52%)
F314	Bipolar affective disorder, current episode severe depression without psychotic symptoms	41 (1.55%)
F315	Bipolar affective disorder, current episode severe depression with psychotic symptoms	29 (1.09%)
F316	Bipolar affective disorder, current episode mixed	137 (5.17%)
F317	Bipolar affective disorder, currently in remission	701 (26.46%)
F318	Other bipolar affective disorders	44 (1.66%)
F319	Bipolar affective disorder, unspecified	380 (14.35%)

### Measures

#### Diagnostic and sociodemographic variables

Firstly, we extracted several diagnostic variables from CRIS including patients’ ICD diagnostic code (F30 or F31) and corresponding recorded diagnostic date. We then extracted several sociodemographic variables including patients’ date of birth, gender, ethnicity, employment status and marital status. Based on this extracted data, age at the time of : 1) diagnosis, 2) data extraction (June 2019) and 3) first relapse, were all computed. The sociodemographic variables were coded as dichotomous variables: gender (male vs female); ethnicity (white vs non-white); marital status (married/in a relationship vs single/divorced/widowed) and employment status (employed vs unemployed/homemaker/retired/student/unable to work).

#### Relapse status

The main outcome was relapse status, which was operationally defined, prior to data extraction, as: (1) admission to inpatient care or a referral to either the home treatment team (HTT), crisis resolution team or liaison psychiatry, in line with previous work on these mental health services (Johnson et al. 2008). Notably, previous bipolar disorder literature outlines the term ‘relapse’ to refer to ‘return of the index episode’ and ‘recurrence’ to capture the ‘occurrence of a new episode’ (Tohen et al. 2009). Thus, for clarity, the definition of relapse adopted within our study would capture both instances of relapse and recurrence. The granularity of the available data precludes us commenting specifically on whether re-emergence refers to relapse or recurrence in each individual case, in accordance with the ISBD taskforce definitions. Relapse data was extracted from structured fields within the CRIS database, to assess for evidence of relapse during the 5-year period (June 2014 to June 2019). To be as accurate as possible, we included any admission/referral to HTT and liaison psychiatry which had a ‘referral reason’ that could be related to a relapse in mental health (e.g. ‘admission’, ‘in crisis’, ‘bipolar disorder’ ‘self-harm’ etc.), whereas others not deemed to be bipolar relapse-related were excluded (e.g. ‘adjustment to health issues’, ‘unexplained physical symptoms’, ‘assessment for physical activity’). Further, to ensure that the admissions/referrals were capturing true instances of relapse, and not any other non-relapse events (e.g. routine medication review etc.), we also then cross-referenced each referral with the patient’s de-identified clinical and assessment notes from within that referral period and systematically searched these notes using a series of six pre-defined keywords: ‘relapse’, ‘deterioration’, ‘unwell’, ‘crisis’, ‘mani*’ and ‘depress*’. Ultimately, this data-cleaning process allowed for the removal of any inappropriate referrals, thus helping to provide a more accurate estimate of BD relapse rate. Relapse status was therefore coded as a dichotomous variable (yes vs no to experiencing at least one relapse episode during the 5-year period).

#### Relapse characteristic variables

Several relapse characteristics were computed (all continuous variables), including the: (1) number of relapses over 5 years for each patient, (2) total number of days between BD diagnosis date and earliest relapse referral/admission date, and (3) total number of days spent in relapse (across all referral periods for everyone), by using the referral/admission start and end dates. To obtain further clinical information about the nature of the relapse episode, the patients’ de-identified clinical notes and care plans from within the referral period were extracted to allow the research team to code for several variables including the: (1) mood episode experienced during relapse (i.e. mania vs depression vs mixed-state), (2) presence of psychotic symptoms (yes vs no) and (3) presence of deliberate self-harm/suicidality or attempts (yes vs no).

#### Clinical history variables

Several pre-defined clinical history characteristics from patients de-identified clinical notes were also extracted. These variables included whether a patient had any reported history of (1) trauma exposure (i.e. emotional/physical/sexual), (2) deliberate self-harm/suicidality and, (3) family history of mental illness (any mental illness), all of which were coded as dichotomous variables (yes vs no to any reported history). This data was extracted from the de-identified patient notes linked to the date of their BD diagnosis assessment. Additionally, this information was also cross-referenced with patients’ future assessments from within the 5-year period, to ensure that any important historic clinical information (that might not have been disclosed at point of initial diagnosis episode) was also captured. Finally, to examine whether the presence of psychotic symptoms was associated with either of our relapse variables, we coded for whether (or not) individuals had either psychotic symptoms reported at diagnosis/relapse and/or whether they had received a schizophreniform disorder diagnosis during the follow-up period. Those who met this criteria (n = 438) were coded as yes to the presence of psychotic symptoms.

### Data analysis

The main outcome was the 5-year rate of relapse, which was calculated by dividing the total number of patients who experienced at least one relapse episode over the 5-year period (June 2014 to June 2019), by the total BD sample. The cohort was split by relapse status (i.e. relapsers vs non-relapsers), and differences in sociodemographic and clinical characteristics were compared*,* with either chi-square (i.e. dichotomous/categorical variables) or independent *t*-tests (i.e. continuous variables). Binary logistic regression analyses were conducted to examine whether any sociodemographic, clinical history or relapse characteristics were longitudinally associated with risk of *any* relapse during the 5-year period.

The outcome variable was relapse status (dichotomous: no vs yes). First, an unadjusted multivariable binary logistic regression analysis was conducted, where a reported trauma history (no vs yes), reported history of deliberate self-harm/suicidality (no vs yes), the presence of comorbid mental health diagnosis (any) (no vs yes) and the presence of psychotic symptoms reported (no vs yes) were entered into the model. These predictor variables were chosen due to them being either being significantly different between the relapse vs non-relapser groups (see Table 2), or to help identify potentially modifiable risk factors associated with relapse status among BD populations. This model was then adjusted to account for several sociodemographic and clinical factors, including age at reported BD diagnosis (continuous), marital status (single/divorced vs in a relationship), employment status (employed vs unemployed) and ethnicity (white vs non-white) (all factors which were significantly different between groups).

**Table 2:** Demographic characteristics for the bipolar cohort (N = 2649) by relapse status

Variables	Non-Relapsers (*n* = 1973)	Relapsers (*n* = 676)	*p *(two-tailed)
BD diagnostic code % (n)^a^			0.66
F30	6.0% (119)	6.5% (44)	–
F31	94.0% (1854)	93.5% (632)	–
Age at BD Diagnosis % (n)^b^			< 0.001
≤ 30 years	17.4% (343)	20.7% (140)	0.16
31–60 years	60.9% (1202)	64.2% (434)	0.32
≥ 61 years	21.7% (428)	15.1% (102)	< 0.001
Age at BD Diagnosis *M* (SD)^c^	47.20 (16.53)	44.33 (15.12)	< 0.001
Gender % (n)^d^			0.16
Male	38.2% (754)	40.1% (271)	–
Female	61.8% (1219)	59.8% (404)	–
Non-binary	0% (0)	0.1% (1)	–
Ethnicity % (n)^e^			0.03
White	68.1% (1328)	65.1% (438)	0.16
Black	9.2% (180)	10.5% (71)	0.32
Asian	18.5% (360)	19.5% (131)	0.55
Mixed	1.8% (36)	3.6% (24)	0.01
Other	2.4% (47)	1.3% (9)	0.09
Marital status % (n)^f^			< 0.001
Single	41.7% (751)	52.1% (332)	< 0.001
Married/Civil partnership	36.7% (660)	24.8% (158)	< 0.001
Divorced/Separated	12.6% (227)	15.2% (97)	0.58
In a relationship	2.9% (53)	3.8% (24)	0.91
Widowed	6.0% (108)	4.1% (26)	0.52
Employment % (n)^g^			< 0.001
Employed (full/part time)	16.7% (36)	11.2% (11)	0.79
Unemployed	30.2% (65)	59.2 (58)	< 0.001
Homemaker	3.3% (7)	1.0% (1)	0.84
Retired	41.4% (89)	22.4% (22)	0.04
Student	3.3% (7)	4.1% (4)	1.00
Unable to work	5.1% (11)	2.0% (2)	0.79
Family history of *any* mental health disorder^h^ % (Yes) (n)	35.4% (698)	41.3% (279)	0.006
Family history of affective disorders (Yes)^h^ % (n)	24.0% (473)	25.1% (170)	0.54
Comorbid diagnoses (Yes) % (n)^i^	15.0% (295)	36.7% (543)	< 0.001
Anxiety disorder (n = 33)	8.5% (25)	3.2% (8)	0.01
Eating disorder (n = 8)	1.0% (3)	2.0% (5)	0.34
Personality disorders (n = 122)	19.3% (57)	26.2% (65)	0.06
PTSD (n = 7)	1.4% (4)	1.2% (3)	0.88
Sleep disorder (n = 5)	0.17% (2)	1.2% (3)	0.52
Substance misuse (n = 307)	44.7% (132)	70.6% (175)	< 0.001
Mixed anxiety/depression (n = 20)	5.1% (15)	2.0% (5)	0.06
Trauma History (Yes) % (n)^j^ (emotional/physical/sexual abuse)	24.5% (483)	40.1% (271)	< 0.001
History of self harm/suicidality at baseline^k^ (Yes) % (n)	18.0% (355)	31.8% (215)	< 0.001

^a^Recorded at baseline; *X*^2^(1) = 0.20, *p* = 0.66

^b^*X*^2^ (2) = 14.91, *p* < 0.001

^c^*t* (2647) = 3.98, *p* <0 .001

^d^*X*^2^ (2) = 3.71, *p* = 0.16

^e^*X*^2^ (4) = 11.03, *p* = 0.03

^f^*X*^2^ (5) = 2035.03, *p* < 0.001

^g^*X*^2^ (6) = 35.14, *p* < 0.001

^h^Family history of any mental health condition: *X*^2^ (1) = 7.52, *p* = 0.006; Family history of affective disorders: *X*^2^ (1) = 0.38, *p* = 0.54

^I^*X*^2^ (1) = 145.95, *p* < 0.001

^j^*X*^2^ (1) = 60.24,* p* < 0.001

^k^*X* (1) = 58.88,* p* < 0 .001

Further, linear regression analyses were conducted to examine factors associated with the number of relapses experienced within the 5-year period, using the number of relapses (continuous) as the outcome variable. In the first model (unadjusted), the predictor variables (reported trauma history, reported history of deliberate self-harm/suicidality, the presence of comorbid mental health diagnosis and the presence of psychotic symptoms reported) were entered. This model was then adjusted, to account for the sociodemographic/clinical factors outlined above. 95% Confidence Intervals (*CIs)* are reported throughout. All the statistical analyses of this study were conducted using SPSS Version 29.

## Results

### Relapse rate

**Fig. 1:**
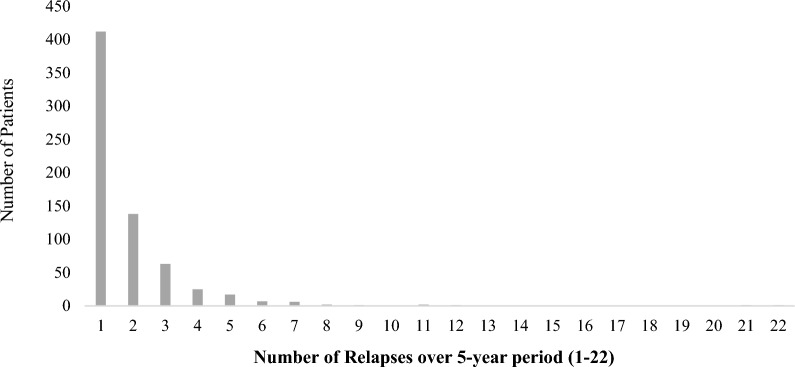
Distribution of the number of the relapses over the 5-year period

The analysed cohort consisted of 2649 patients, aged ≥ 18 years old, and with a diagnosis of BD (ICD codes F30/F31) as of June 2014. The mean age of the cohort was 54.26 years old (*SD* = 16.31), and the majority were coded as female (61.30%). From this sample, there was evidence of 1248 relapse instances within the 5-year period. These relapses then equated to 676 individual patients in total (as some individuals had experienced multiple relapse referrals) who had experienced *at least one episode of relapse* within the 5-year period (i.e. *relapsers*). Thus, based on this data, the 5-year relapse rate for those who had experienced *at least one relapse* was calculated at 25.52%. More specifically, out of the total sample, 15.55% (n = 412) experienced one relapse episode, whereas 9.97% (n = 264) experienced multiple relapses (i.e. at least 2) (see Fig. 1). From this point on, patients who had experienced at least one episode of relapse are referred to as *relapsers* (i.e. n = 676), whereas those who did not experience a relapse are referred to as *non-relapsers* (i.e. n = 1973). Lastly, from the baseline sample, 7.2% (n = 190) of BD patients were reported to have died during the 5-year period (causes of death unknown). Based on available data around patient’s deaths, the mean age of death was calculated to be 69.19 (*SD* = 16.43; Mdn = 71) and the mean time (years) from age at earliest reported BD diagnosis to death was 4.49 (*SD* = 2.43).

### Sociodemographic data by relapse status

Table 2 provides an overview of the demographic characteristics of the non-relapsers (n = 1973) vs relapsers (n = 676). Compared to the non-relapsers, relapsers in this sample were significantly more likely to be of younger age, be single/un-married, unemployed and of mixed ethnicity. There were no significant differences found in BD diagnostic code or gender linked to relapse status. Those who relapsed were also significantly more likely to have reported a history of previous self-harm/suicidality and prior exposure to traumatic events (e.g. sexual, physical, emotional abuse) at diagnosis assessment, compared to non-relapsers. Lastly, over the 5-year period (June 2014 to June 2019), relapsers were significantly more likely to have comorbid mental health diagnoses.

### Relapser sample characteristics

Figure 1 outlines the distribution of relapses over the 5-year period for the relapse sample (N = 676) and demonstrates that 60.9% of the relapser sample had experienced one relapse during the 5-year period, with the remainder (39.1%) having experienced multiple relapses ranging from 2 to 22. The mean number of relapses recorded over the 5-year period was 1.85 (*SD* = 1.77).

Table 3 examines the clinical characteristics of the relapse cohort and relapse referrals. Most relapser patients fell within the 31–60 years old age bracket at the time of their first recorded relapse. It appeared that patients experienced their first relapse within approximately 5 years of their recorded BD diagnosis date (Mdn = 1840 days). The mean total time spent in relapse (across all relapse periods per patient) was 24.59 (*SD* = 33.72) days. There was an equal split between the type of mood episodes (i.e. mania vs depression) associated with each relapse. Further, 13.3% of the referrals reported psychotic symptoms at point of relapse. Of those referrals in which deliberate self-harm/suicidality was a factor, there was a higher proportion of reported suicidal ideation at relapse, vs suicidal attempts and deliberate self-harm.

**Table 3:** Descriptive statistics for the patients who relapsed (n = 676; i.e. subject-based) and for the relapse referrals (n = 1248; relapse-based)

Variables	% (n)
**Subject-based** **analyses** **(n = 676)**	
Age at first relapse % (n)	
≤ 30 years old	11.4% (77)
31–60 years	63.3% (428)
≥ 61 years old	25.3% (171)
Age at first relapse *M* (SD)	49.71 (15.48)
Number of relapses over 5 years *M* (SD); *Range*	1.85 (1.77); *1–22*
Time (days) between recorded BD diagnosis and first recorded relapse *Mdn; Range*	1840.50; *76–14,126*
Time (days) spent in relapse over the 5-year period *M* (SD)	24.59 (33.72)
Mood episode at first relapse episode % (n)	
(Hypo) Mania	45.1% (305)
Depression	38.9% (263)
Mixed	6.5% (44)
Unclear^a^	9.0%(61)
Non-BD-Relapse^b^	0.4% (3)
**Relapse-based analyses** **(n = 1248)**	
Proportion of relapse referrals (n = 1248) by mood episode overall % (n)	
(Hypo)Mania	42.7% (533)
Depression	42.% (524)
Mixed	7.2% (90)
Unclear^a^	7.7% (96)
Non-BD-Relapse^b^	0.4% (5)
Proportion of relapse referrals (n = 1248) that involved reported	
Deliberate self-harm reported to be present % (n)	1.0% (13)
Suicidal ideation reported to be present % (n)	11.6% (145)
Suicide attempt reported to be present % (n)	6.1% (76)
Self-harm/suicidal ideation/attempt reported to not be present	38.6% (482)
Presence of self-harm/suicidal ideation/attempt unknown^c^	42.6% (532)
Proportion of relapse referrals (n = 1248) where	
Psychotic symptoms reported to be present	13.3% (166)
Psychotic symptoms reported not to be present	42.8% (534)
Presence of psychotic symptoms unknown^c^	43.9% (548)
Prescribed Medication at Time of each Relapse Referral (n = 1248)	
Antidepressants	31.0% (387)
Antipsychotics	69.6% (869)
Mood Stabilisers/Anticonvulsants	46.3% (578)

^a^Here, it was clear the patient had experienced a relapse in their BD, but the specific type of mood episode was unclear based on the notes

^b^Notes here focussed on a relapse in patients’ comorbid diagnoses (e.g. eating disorder) rather than explicitly commenting on a BD relapse

^c^Here, it was unknown as there was no reference to the presence (or non-presence) of deliberate self-harm/ suicidal ideation/attempt or psychotic symptoms made in notes

### Factors associated with relapse status (any relapse over the 5-year period)

The adjusted model accounted for 27.8% of the variance (Nagelkerke *R*^*2*^). The model showed that a reported history of reported self-harm/suicidality (OR 2.17, *CI* 1.15–4.10, *p* = 0.02) the presence of a comorbid mental health diagnosis (OR 2.59, *CI* 1.35–4.97, *p* = 0.004) and the presence of psychotic symptoms (OR 3.66, *CI* 1.89–7.08, *p* < 0.001) were all factors significantly associated with relapse status.

### Factors associated with the number of relapses over the 5-year period

The adjusted model accounted for 18.1% of the variance, and results showed that a reported history of trauma (*β* = 0.51, *CI*  0.07–0.95, *p* = 0.03), self-harm/suicidality (*β* = 0.69, *CI*  0.21–1.17, *p* = 0.005), the presence of psychotic symptoms (*β* = 1.05, *CI* 0.55–1.56, *p* < 0.001), comorbidity (*β* = 0.52, *CI* 0.07–1.03, *p* = 0.047) and ethnicity (*β* =− 0.44, *CI* − 0.87 to − 0.003, *p* = 0.048) were all significantly associated with the number of relapses.

## Discussion

This study, as far as the authors are aware, is the largest study of BD patients (N = 2649) within the UK to estimate the rate of BD relapse over a 5-year period. The rate of relapse in this study was 25.52% over a 5-year period. This rate is comparable to previous naturalistic studies (please see Additional file 1) that have assessed BD relapse rates, such as the US-based, STEP-BD study (Perlis et al. 2006), which calculated a 1-year relapse rate of 48.5% and in Vázquez et al.' s (2015) review paper (Vázquez et al. 2015), which estimated recurrence rates of 26.3% among the naturalistic studies (n = 10) reviewed.

To date, O’Hagan and colleagues (2017) appears to be the only naturalistic UK study to estimate BD relapse rate. Compared to our current study, O’Hagan et al.'s (2017) study noted slightly higher rates of recorded substance misuse (14.1% vs 11.5% in the present study; please see Additional file 1), which may have increased the rate of bipolar relapse they found—i.e. increased rates of substance misuse may have escalated the deterioration of a person’s mental health leading to additional hospitalisation. Another possibility is that our data may be underestimating the true rate of relapse for people with BD residing in Birmingham. For instance, it is possible that not all instances of BD relapse may have precipitated an admission to hospital or a referral to crisis services. Indeed, Hong et al.’s (2010) study reported that only 60% of relapses were re-admissions to hospital. Community mental health services may have managed and attenuated relapses, as opposed to sparking additional referrals to crisis teams/ inpatient admissions. Our study therefore adds value by exploring relapse (normally defined in research studies as hospitalisation) within a routine healthcare system, and with relapse being clinician assessed.

Further, a striking proportion of deaths were reported in our study. Out of the 2649 patients at baseline, 7.2% of this sample (n = 190) had died during the 5-year follow-up; a figure which supports findings from previous BD cohort studies (Hayes et al. 2017). Based on these figures, the death rate among BD populations appears to be higher than the death rate among populations with other serious mental health conditions, such as treatment-resistant depression and schizophrenia, which appear to be around 5.05% (e.g. Madsen et al. 2021) and 2.7–4.1% (Kurdyak et al. 2021), respectively. BD patients consistently show an average reduced lifespan compared to the general population (Staudt Hansen et al. 2019) and our findings further highlight the need for better treatment for this neglected population.

The second aim of this study was to examine the potential factors associated with relapse status and the number of relapses during the 5-year period. Results showed that after controlling for several sociodemographic variables, a reported history of deliberate self-harm/suicidality, having a comorbid mental health diagnosis and the presence of psychotic symptoms, at either first recorded diagnosis or relapse, were all significantly associated with experiencing a relapse episode over the 5-year period (i.e. at least one relapse). Further, when examining factors associated with the *number* of relapses over the 5-year period, a reported history of deliberate self-harm/suicidality, trauma, the presence of psychotic symptoms (at either first recorded diagnosis or relapse), comorbidity and ethnicity, were all significantly associated with a higher number of relapses during the 5-year period, suggesting the robustness of our findings.

Combined, these findings highlight four potentially modifiable targets for future BD interventions centred on reducing: (1) rates of self-harm/suicidality, (2) the damaging effects of trauma, (3) rates of mental health comorbidity and (4) targeting the reduction of psychotic features within BD. These current results support a plethora of research highlighting the high prevalence of self-harm/suicidality (Clements et al. 2013) and psychiatric comorbidity, particularly anxiety disorders (Simon et al. 2004), among people with BD. Comorbid anxiety disorders specifically pose a unique treatment challenge for BD patients, given that SSRI antidepressants and others used to treat anxiety, can increase the risk of relapse in BD. Similarly, there are high levels of comorbidity between BD and borderline personality disorder (Durdurak et al. 2022) and attention deficit hyperactivity disorder (Schiweck et al., 2021). Other than pharmacological treatment or disorder specific psychological therapy, another approach to combating the high rates of comorbidity present in BD, and potentially helping to reduce relapse rates, is to adopt transdiagnostic treatment protocols for BD patients. Early feasibility trials assessing transdiagnostic approaches show promising results (Perich et al. 2020), however, further research, such as RCTs, are needed to better determine their effectiveness. Network analyses, which provides centrality statistics that indicate how variables are related to others in the network, may also be useful for BD given that symptoms overlap with various other psychiatric disorders (Scott et al. 2021). Further, within BD cases, the presence of psychotic symptoms is a common phenomenon (Dell’Osso et al. 2017) and has previously been identified as a potential risk factor for bipolar relapse (Carlson et al. 2012), as it likely represents a more complex and severe form of bipolar (Elowe et al. 2022).

A burgeoning area of research demonstrates the significant role that trauma exposure, particularly during childhood, plays in the onset and trajectory of BD (Aas et al. 2016). Agnew-Blais and Daneses’ (2016) meta-analysis (Agnew-Blais and Danese 2016) shows that the course of BD is more pernicious and complex when individuals have been exposed to childhood trauma. This has been observed by markers such as an earlier age BD onset, the presence of psychotic features, cognitive impairment, rapid cycling, suicidality, greater depressive symptoms, poorer global functioning, increased number of mood episodes, greater comorbidity, and a poorer response to front-line biological treatments, such as lithium (Aas et al. 2016). Thus, the idea of trauma-focussed interventions for BD patients is gaining traction (Hett et al. 2022), and this current study further supports this idea by showing that trauma exposure in BD patients is also associated with relapse outcomes.

### Strengths and limitations

This study has several strengths, the first being its large sample size. Additionally, unlike in RCTs, the naturalistic data here arguably provides a more clinically valid account of relapse rates, as it includes BD patients with more complex presentations (e.g. comorbid diagnoses) that are routinely seen in secondary care. It is also important to point out that our data is capturing clinician-defined relapse, and that all data used has been entered and completed by healthcare professionals, thus providing an invaluable insight into the service provision and journey to recovery (or otherwise) experienced by patients with BD. However, there are some limitations to note. Within the UK and the NHS healthcare system, the treatment of BD, as with other complex and severe forms of mental illness, routinely falls under secondary care mental health services, especially for people with more severe forms of the condition. However, being provided treatment within secondary care does not exclude management within primary care. A proportion of BD cases may either first present themselves within primary care (e.g., via GP appointment) or indeed, following discharge from secondary care services, those with BD may prefer to have ongoing medication management within primary care (NICE 2022). Secondary mental healthcare management is the predominant option for those with BD who are more unwell, and as a result, our data may be biased towards that group, and not be generalisable to people with more milder forms of BD. By default, our methods used a calendar-based approach to cohort definition, meaning that data is available on large numbers of patients who have a diagnosis of BD and are receiving routine care within secondary mental health services. This provides much needed valid data on the progress and outcomes of people with BD and avoids the well-known issues of lack of generalisability of research cohorts. The limitations of using our electronic health records approach, is that data on variables that could be of interest to modelling, such as presentation on index episode, are not reliably available, and our results should be considered in this light. As the analyses are based on electronic health records within an NHS Trust, we can only analyse data that has been recorded and documented. For instance, to obtain a BD diagnosis date in this study, we selected the earliest recorded bipolar diagnosis date for each patient. However, as outlined by the data in Table 1, it is likely that patients may have received their true BD prior to this date, and if so, this would skew the time from diagnosis to relapse variable computed in this study. Some other important associations/predictors of relapse were inevitably not available in the data, such as cognitive impairments (Miskowiak et al. 2022) or early age illness onset (Davarinejad et al. 2021). Further, whilst the data has strong ecological validity, there may be differences with studies that have used research assessments. We would view these different approaches as providing complimentary data. Finally, the available data does not include information from some mental health services providing care to young people under 25 years, this being provided by an organisation from which we could not obtain data. As such, our data does not include information on crisis team presentations and some inpatient admission. Therefore, the relapse rate found may be an underestimate of the total BD population. However, although the rate of paediatric BD diagnosis has doubled in outpatient clinical settings (Meter et al. 2011), the peak incidence for most people with BD is 25 years and over (Bellivier et al. 2003), and there is a well-recognised diagnostic delay of 8–10 years; so the extent of our underestimate is difficult to know. Our results are most valid for people who are over the age of 25 years.

### Future research

Given the benefits linked to using naturalistic data such as electronic health records, future research may benefit from adopting a machine learning approach to better predict who may relapse and why. Recent work has utilised machine learning approaches to pinpoint important predictors to depressive relapses in BD (Siqueira et al. 2021) using the STEP-BD dataset. However, replicating this approach within a large UK mental health Trust would be advantageous to better determine relapse risk factors and aid decision-making in the treatment and prevention of BD, specifically within the UK. Additionally, future research would benefit from further examining the comorbidity of physical health conditions within individuals diagnosed with BD to better understand the treatment needs and outcomes of this group and offer better insight to healthcare professionals on ways to tailor treatment approaches for this patient group.

## Conclusions

In a non-selected retrospective cohort study of 2649 people with BD receiving secondary mental health services in the UK, around 1 in 4 people relapsed over a 5-year period, with approximately 40% of this relapser sample experiencing more than one relapse. Interventions targeting the impacts of trauma, suicidality, comorbidity and psychotic features, could help to prevent relapse in people with BD and should be considered in relapse prevention plans.

## Supplementary Information

**Additional file 1: Table S1.** Overview of naturalistic studies reporting BD relapse rates.

## Data Availability

The de-identified data are not publicly available due to the confidential nature of the data. CRIS was developed with extensive involvement from service users and adheres to strict governance frameworks managed by service users. The data are de-identified and used in a data secure format and all service users within the Trust have the choice to opt-out of their anonymised data being used. Approval for data access can only be provided by the Research and Innovation Department at Birmingham and Solihull Mental Health Foundation Trust (BSMHFT).
