# Supplementary material for: Rates and associations of relapse over 5 years of 2649 people with bipolar disorder: a retrospective UK cohort study
Source: Int J Bipolar Disord. 2023 Jun 30;11:23. doi: 10.1186/s40345-023-00302-x (PMC10313572; doi:10.1186/s40345-023-00302-x)
Supplement: Supplementary file 1 — Additional file 1: Table S1. Overview of naturalistic studies reporting BD relapse rates. [file 40345_2023_302_MOESM1_ESM.docx]

**Table 1Additional file materials**: Overview of naturalistic studies reporting BD relapse rates

**Highlighted text represents studies which utilised similar relapse definitions to the one adopted to our current study.*

| **Study** | **Relapse Rate** | **Country** | **Sample Information** | **Relapse definition/ methods information** |
| --- | --- | --- | --- | --- |
| Our current study | 5-year: 25.5% | UK | - **N =** 2649 (676 relapsed) - **Female (%):** 61.26% - **Suicidal Ideation reported (%):** 21.52% - **Substance Misuse (%):** 11.5% | - Clinician-defined relapse; determined by hospitalization/ referrals to acute mental health crisis. |
| Coryell et al., 1989  (NIMH Psychobiology of depression study) | 5-year: 67.5% (at least one episode of relapse)  Hospitalisation specific =95% | US | - **N = 442** - Bipolar sample was 117 (split as BD1 vs BD2 and 79 individuals had at least one relapse. - Non diverse sample – all White – English was primary spoken language. | - Relapse required reappearance of symptoms to meet criteria for the disorder. - Relapse assumed that there was a prior recovery period of at least 8 weeks. |
| Hong et al. 2010  (EMBLEM study) | 2-year: 54.3% | 14 European countries, including UK (but UK sample size not reported in the paper) | - **N =** 1379 - **Reported BD onset age (Mean)** = 36.2 - **Female (%)** = 39.2% - **Suicidal ideation (%):** 27% - **Substance misuse (%):** 3.6% | - Defined it as point increase in the clinical global impression BD scale, **inpatient admission** or relapse determined after psychiatric assessment. |
| Li et al. 2018 | 6-year: 77.6% | Taiwan | - **N = 165** - **Reported BD onset Age (Mean) =** 27.3 - **Female (%)** = 52.1% - **Suicidal Ideation (%)** = 23% - **Substance misuse (%)** = 33.3% | - Rehospitalisation to one hospital |
| O’Hagan et al. 2017 | 1-year: 32.2% | UK | - **N =** 516 - **Female (%):** 56.4% - **Suicidal Ideation reported (%):**  Not reported - **Substance Misuse (%):** 14.1% - **Reported BD onset age (Mean):** 43.8 | - Relapse was assessed as readmission/ rehospitalisation **to one single mental health unit** |
| Perlis et al. 2006  (STEP-BD) | 2-year: 48.5% | US | - N = 858 - **Reported BD onset age (Mean):** 16.7 - **Female (%):** 59% - **Suicidal ideation (%):** 35.6% - **Substance misuse (%):** 49.3% | - Recurrence was defined as meeting the full DSM-IV criteria for a manic, hypomanic, mixed, or depressive episode on any one follow-up visit. Subsyndromal mood symptoms were defined as the presence of more than two syndromal features of either depression, mania, or hypomania without meeting the full DSM-IV criteria for a mood episode. Occurrence of subsyndromal mood symptoms during follow-up was not considered a recurrence. |
